# MCPIP1 Enhances TNF-α-Mediated Apoptosis through Downregulation of the NF-κB/cFLIP Axis

**DOI:** 10.3390/biology10070655

**Published:** 2021-07-12

**Authors:** Fat-Moon Suk, Chi-Ching Chang, Pei-Chi Sun, Wei-Ting Ke, Chia-Chen Chung, Kun-Lin Lee, Tze-Sian Chan, Yu-Chih Liang

**Affiliations:** 1 Division of Gastroenterology, Department of Internal Medicine, Wan Fang Hospital, Taipei Medical University, Taipei 11696, Taiwan; 2 Department of Internal Medicine, School of Medicine, College of Medicine, Taipei Medical University, Taipei 11031, Taiwan; fmsuk@tmu.edu.tw (F.-M.S.); ccchang@tmu.edu.tw (C.-C.C.); fzesian@tmu.edu.tw (T.-S.C.); 3 Division of Rheumatology, Immunology and Allergy, Taipei Medical University Hospital, Taipei 11031, Taiwan; 4 School of Medical Laboratory Science and Biotechnology, College of Medical Science and Technology, Taipei Medical University, Taipei 11031, Taiwan; m120098047@tmu.edu.tw (P.-C.S.); b104094051@tmu.edu.tw (W.-T.K.); shettoangel@gmail.com (C.-C.C.); kunlinleetw@gmail.com (K.-L.L.); 5 Ph.D. Program in Medical Biotechnology, College of Medical Science and Technology, Taipei Medical University, Taipei 11031, Taiwan; 6 Traditional Herbal Medicine Research Center, Taipei Medical University Hospital, Taipei 11031, Taiwan

**Keywords:** MCPIP1, TNF-α, apoptosis, caspase-1, importin

## Abstract

**Simple Summary:**

TNF-α is considered to be a potential therapeutic drug for cancer, but the systemic toxicity problem still exists in clinical use. MCPIP1 plays a crucial role in anti-inflammatory and anti-viral contexts, as well as in the inhibition of cell growth. This study aims to investigate the roles of MCPIP1 in TNF-α-treated cells and their underlying molecular mechanisms. MCPIP1 was found to enhance TNF-α-induced apoptosis by activating the caspase cascade, and pan-caspase inhibitor and the caspase-1/-4 inhibitor could reverse TNF-α/MCPIP1-mediated apoptosis. MCPIP1 was first identified with cleavage site of caspase-1/-4 and could be cleaved during apoptosis. Downregulation of NF-κB activation and a caspase-8 inhibitor, cFLIP, were associated with TNF-α/MCPIP1-mediated apoptosis.

**Abstract:**

Monocyte chemoattractant protein-1-induced protein 1 (MCPIP1) is rapidly produced under proinflammatory stimuli, thereby feeding back to downregulate excessive inflammation. In this study, we used the stable, inducible expressions of wild-type (WT) MCPIP1 and an MCPIP1-D141N mutant in T-REx-293 cells by means of a tetracycline on (Tet-on) system. We found that WT MCPIP1 but not MCPIP1-D141N mutant expression dramatically increased apoptosis, caspase-3, -7, -8, and -9 activation, and c-Jun N-terminal kinase (JNK) phosphorylation in TNF-α-treated cells. The pan-caspase inhibitor, z-VAD-fmk, and the caspase-1 inhibitor, z-YVAD-fmk, but not the JNK inhibitor, SP600125, significantly reversed apoptosis and caspase activation in TNF-α/MCPIP1-treated cells. Surprisingly, MCPIP1 itself was also cleaved, and the cleavage was suppressed by treatment with the pan-caspase inhibitor and caspase-1 inhibitor. Moreover, MCPIP1 was found to contain a caspase-1/-4 consensus recognition sequence located in residues 234~238. As expected, the WT MCPIP1 but not the MCPIP1-D141N mutant suppressed NF-κB activation, as evidenced by inhibition of IκB kinase (IKK) phosphorylation and IκB degradation using Western blotting, IKK activity using in vitro kinase activity, and NF-κB translocation to nuclei using an immunofluorescence assay. Interestingly, MCPIP1 also significantly inhibited importin α3 and importin α4 expressions, which are major nuclear transporter receptors for NF-κB. Inhibition of NF-κB activation further downregulated expression of the caspase-8 inhibitor, cFLIP. In summary, the results suggest that MCPIP1 could enhance the TNF-α-induced apoptotic pathway through decreasing NF-κB activation and cFLIP expression.

## 1. Introduction

Tumor necrosis factor (TNF)-α is a multifunctional cytokine that binds to the TNF receptor (TNFR) and transduces cell survival, apoptosis and necroptosis signals. TNF-α is also involved in various diseases, such as bone loss, septic shock, rheumatoid arthritis, and other inflammatory diseases [1]. After TNF-α binds to its receptor, TNFR1, two protein complexes can subsequently be formed. The first is called the TNFR1 signaling complex (TNF-RSC, also known as complex-I), which is related to NF-κB’s transcriptional activity and cell survival; the second complex is called complex-II and is related to apoptosis [2]. Generally, the signal pathway induced by complex-I can inhibit the apoptotic signal pathway of complex-II. Members of complex-I include TNFR1-associated death domain protein (TRADD), receptor-interacting protein kinase (RIP1), TNFR-associated factor 2 (TRAF2) or TRAF5, and the cellular inhibitor of apoptosis protein 1 and 2 (cIAP1 and cIAP2). The cIAP is a ubiquitin E3 ligase, which can catalyze K63-linked polyubiquitination of RIP1 [3,4]. The polyubiquitination of RIP1 then attracts transforming growth factor (TGF)-β-activated kinase 1 (TAK1) and IκB kinase (IKK) complexes to bind to complex-I, thus activating TAK1 and IKK. TAK1 activates the c-Jun N-terminal kinase (JNK) signal pathway, and IKK phosphorylates IκB, which then can be ubiquitinated by Skp1-Cullin-F-box protein β-transducin repeat-containing protein (SCF^β-TrCP^, an E3 ligase), and it is finally degraded by a proteasome, thereby activating NF-κB.

After complex-I is activated, TNFR1 is then internalized and forms complex-II in the cytoplasm. Complex-II contains TRADD, TRAF2, Fas-associated death domain (FADD), and caspase-8. Caspase-8 then dissociates from complex-II, cleaves Bid, and activates the mitochondrial apoptotic pathway (caspase-9-dependent activation of caspases-3, -6, and -7) to initiate cell apoptosis [5]. On the contrary, activated NF-κB from complex-I can induce expressions of antiapoptotic molecules, such as IAPs and cFLIP [6], which are able to inhibit caspase-8 induced by complex-II. In addition, complex-I also binds to pro-caspase-1 and then promotes caspase-1 activation [7]. Since many studies have demonstrated that inflammatory caspases engage in crosstalk with apoptotic caspases [8], activated caspase-1 may, in turn, activate caspases-3, -6, and -7, and activated caspases-8 and -9 may also further activate caspase-1 [9,10]. Moreover, deubiquitinated RIP1 can dissociate from complex I and assemble into complex II, and then initiate caspase-independent necrotic cell death (necroptosis) [11]. Therefore, the balance of the TNF-α-induced complex-I and complex-II signaling pathways affects the final fate of cells [12].

Monocyte chemoattractant protein (MCP)-1-induced protein 1 (MCPIP1, also known as Zc3h12a and regnase-1) was first discovered to be induced by MCP-1 in human monocytes. MCPIP1 is a member of CCCH (C3H)-type zinc finger (ZF) protein family, containing C3H-type ZF, Nedd4-BP1, YacP nuclease/deubiquitinase (NYN/DUB), and proline-rich (PRR) domains [13]. It is induced during the inflammation process of macrophages, and negatively feeds back to regulate the production of proinflammatory cytokines and inducible nitric oxide synthase (iNOS) induced by lipopolysaccharide (LPS). Therefore, MCPIP1 plays an important role in the downregulation of immune inflammatory responses [14,15]. Because MCPIP1 has a ZF domain, it can bind to both DNA and RNA molecules to regulate gene transcription and RNA metabolism, respectively. When MCPIP1 acts as a transcription factor, it can regulate expressions of apoptosis- and angiogenesis-related genes [16]. It can also bind to the 3′ untranslated region (3′UTR) of messenger (m)RNAs, such as interleukin (IL)-1β, IL-6, IL-8, IL-12, and IL-17 mRNAs [17], and results in degradation of these mRNAs by its ribonuclease (RNase) activity. However, unlike other C3H ZF proteins, MCPIP1 binds to stem-loop (SL) structures of mRNA instead of AU-rich elements (AREs) [18]. In addition, MCPIP1 also recognizes the SL structure of human and viral pre-micro (mi)RNAs, and cleaves pre-miRNAs [19]. Our own and other previous studies found that MCPIP1 exhibited antiviral activity through the decreasing of viral RNA levels and the prevention of viral infection, such as hepatitis C virus (HCV) and human immunodeficiency virus (HIV)-1 infection [13,20,21]. The deubiquitinase activity of the NYN domain of MCPIP1 can remove the K63-linked polyubiquitin chain of TRAF2, TRAF3, and TRAF6 in complex-I, thereby inhibiting TNF-α-induced survival signaling pathways, including JNK and NF-κB signals [22]. In addition, MCPIP1 recruits ubiquitin-specific protease 10 (USP10), which then cleaves the K48-linked linear polyubiquitin chain of the NF-κB essential modulator (NEMO), thereby inhibiting activation of genotoxic NF-κB [23].

Recent studies reported that MCPIP1 has anti-inflammatory activities and promotes cell apoptosis. Therefore, this study used the Tet-on system to overexpress wild-type (WT) MCPIP1 and the MCPIP1-D141N mutant, to explore whether MCPIP1 expression affects the action of TNF-α. Our results showed that MCPIP1 inhibited the induced NF-κB survival pathway, thus enhancing the apoptosis pathway in TNF-α-treated cells. We also found for the first time that MCPIP1 had caspase-1/-4 consensus recognition sequences and could be cleaved by caspases-1/-4, and that MCPIP1 inhibited both importin α3 and importin α4 and resulted in a decrease in the nuclear translocation of NF-κB.

## 2. Materials and Methods

### 2.1. Cell Lines and Antibodies

Human embryonic kidney T-REx-293/hemagglutinin (HA)-MCPIP1 and T-REx-293/HA-MCPIP1-D141N cell lines were kindly provided by Dr. Yi-Ling Lin (Institute of Biomedical Sciences, Academia Sinica, Taipei, Taiwan). The cells can be induced by tetracycline (Tet) to express HA-MCPIP1 or HA-MCPIP1-D141N and were cultured in Dulbecco’s modified Eagle medium (DMEM) containing 10% fetal bovine serum (FBS). Anti-cleaved caspases-3, -7, -8, and -9 as well as anti-phospho-extracellular signal-regulated kinase (ERK), anti-phospho-JNK, anti-phospho-p38, anti-phospho-IκBα, anti-phospho-IKKα/β, anti-IκBα, anti-cFLIP, anti-cIAP1, anti-HA, and anti-poly(ADP ribose) polymerase (PARP) antibodies were purchased from Cell Signaling Technology (Beverly, MA, USA); the anti-GAPDH, anti-KPNA3, anti-KPNA4, and anti-MCPIP1 antibodies were obtained from GeneTex International (Hsinchu City, Taiwan); the anti-IKKα/β, anti-NF-κB p50, and anti-NF-κB p65 antibodies, and Protein A/G PLUS-Agarose were purchased from Santa Cruz Biotechnology (Santa Cruz, CA, USA); and the anti-α-tubulin antibody was obtained from Sigma-Aldrich (St. Louis, MO, USA).

### 2.2. Cell Viability Assay

Viable cells were measured using a 3-(4,5-dimethyl-2-thiazolyl)-2,5 diphenyl-2H-tetrazolium bromide (MTT, Sigma Chemical, St. Louis, MO, USA) assay. Briefly, cells were washed with phosphate-buffered saline (PBS) and incubated with MTT, and dimethyl sulfoxide (DMSO) was added to extract the intracellular MTT dye as described previously [24]. The absorbance of the extracted dye was measured at 540 nm by an enzyme-linked immunosorbent assay (ELISA) reader.

### 2.3. Flow Cytometry

Cells were harvested by trypsin-EDTA solution, collected in PBS, and stained with propidium iodide (PI) (Sigma-Aldrich) at room temperature for 15 min in the dark, and then the cell cycle distribution was analyzed by FACScan flow cytometry using CellQuest 3.3 analytical software (Becton Dickinson, San Jose, CA, USA) as described previously [25].

### 2.4. Western Blotting

Total cellular proteins were collected in Golden lysis buffer, and resolved using sodium dodecylsulfate-polyacrylamide gel electrophoresis (SDS-PAGE) as described previously [26]. Proteins in the gel were transferred onto polyvinylidene difluoride (PVDF) membranes (Amersham, Arlington, IL, USA), blocked in 1% bovine serum albumin (BSA), and then incubated with specific primary antibodies and secondary antibodies conjugated to horseradish peroxidase (HRP). HRP activities of antigen–antibody complexes were detected using an enhanced chemiluminescence (ECL, Thermo Fisher Scientific Taiwan, Taipei, Taiwan) kit in an ImageQuant LAS 4000 Biomolecular Imager (GE Healthcare Life Sciences, Marlborough, MA, USA).

### 2.5. Real-Time Reverse-Transcription Polymerase Chain Reaction (RT-PCR)

Total RNA was extracted with TRI reagent (Sigma-Aldrich) and the mRNA was reverse-transcribed into complementary (c)DNA and amplified using a GoScript^TM^ Reverse Transcription System (Promega, Madison, WI, USA) and a SuperTaq DNA Polymerase kit (Ambion, Austin, TX, USA), respectively, according to the manufacturer’s instructions. Sequences of the oligonucleotide primers were 5′-GCTCAGCCACCAGGAAGTTA-3′ and 5′-GCTGGGAAGTGTGAAAGAGC-3′ for importin α3; 5′-GTCCTTCATCAGTCCCTCCA-3′ and 5′-AGCTGGCAAAATCTCCTGAA-3′ for importin α4; and 5′-TGAAGGTCGGTGTGAACGGATTTGGC-3′ and 5′-CATGTAGGCCATGAGGTCCACCAC-3′ for GAPDH. PCR conditions were as follows: 5 min at 94 °C, followed by 30 cycles of 1 min at 94 °C, 1 min at 52 °C, and 1 min at 72 °C, with a final step at 72 °C for 10 min. The amplified cDNA was separated on 1.2% agarose gels and stained with SYBR Green dye [27].

### 2.6. Immunofluorescence (IF) Assay

The cells were washed with PBS and fixed using 4% paraformaldehyde in PBS pH 7.4. After fixation, the cells were permeabilized in PBS with 0.2% Triton X-100, 0.05% Tween 20, and 0.3% BSA, and incubated with the following reagents: blocking solution (5% BSA), anti-NF-κB p65 primary antibody, biotinylated anti-rabbit secondary antibody, and Alexa Fluor™ 568 streptavidin (ThermoFisher Scientific Taiwan, Taipei, Taiwan). 4′,6-Diamidino-2-phenylindole (DAPI) was used to stain nuclei [24], and Prolong Gold Antifade Mountant (Thermo Fisher Scientific Taiwan) was applied to mounted cells. Fluorescence was observed and photographed using the TCS SP5 Confocal Spectral Microscope Imaging System (Leica Microsystems, Singapore).

### 2.7. In Vitro Kinase Activity Assay

Total cellular proteins were harvested in Golden lysis buffer, and IKKα was immunoprecipitated with an IKK-α-specific antibody and protein A/G-PLUS agarose [28]. The precipitates were used for determining the IKK kinase activity through incubation with 5 μM cold ATP, 10 μCi [γ-^32^P] ATP (5000 Ci/mmol, Amersham, The Netherlands), and 1 μg GST-IκBα fusion protein (Santa Cruz Biotechnology) as the substrate in the kinase buffer. The reaction was stopped by adding Laemmle’s loading buffer and heating for 10 min at 100 °C. Then, samples were resolved using 8% SDS-PAGE and the gels were dried. The substrate bands were visualized by autoradiography and quantitated by densitometry (IS-1000 Digital Imaging System, Alpha Innotech Crop., San Leandro, CA, USA).

### 2.8. Electrophoretic Mobility Shift Assay (EMSA)

The nuclear proteins and ^32^P-labeled double-stranded NF-κB oligonucleotide probe were prepared as described previously [28]. Briefly, nuclear proteins were incubated with the probe at room temperature for 20 min, and the probe/protein complex was separated on 6% non-denaturing acrylamide gels. After electrophoresis, the gels were dried by dry-heat vacuum method and the bands were visualized by autoradiography. Sequences of the WT and mutant NF-κB oligonucleotides were 5′-AGTTGAGGGGACTTTCCCAGGC-3′ and 5′-AGTTGAGGGGACTTTCCCAGGC-3′, respectively (the consensus sequence and binding site for NF-κB complexes are underlined).

### 2.9. Statistical Analysis

All data were analyzed by one-way ANOVA followed by Tukey’s post hoc test, and differences were considered significant at *p* < 0.05.

## 3. Results

### 3.1. MCPIP1 Enhanced the Sub-G_1_ Population and Activation of the Caspase Cascade

**Figure 1:**
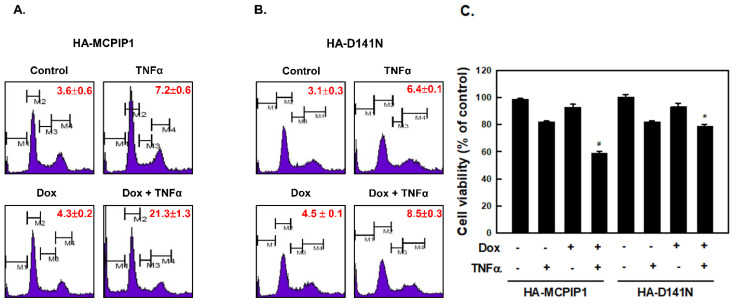
Overexpression of MCPIP1 enhanced the TNF-α-induced sub-G1 population. T-REx-293 cells with (**A**,**C**) wild-type hemagglutinin (HA)-MCPIP1 or (**B**,**C**) the HA-MCPIP1-D141N mutant (HA-D141N) were pretreated without or with 1 μg/mL of doxycycline (Dox) for 16 h, and then treated with 10 ng/mL TNF-α for 2 h. (**A**,**B**) The cell cycle distribution was determined by FACS using propidium iodide staining, and each percentage (%) of sub-G1 population is shown, and (**C**) the cell viability was analyzed using an MTT method. M1, sub-G1 population; M2, G0/G1 population; M3, S population; M4, G2/M population. Each value is presented as the mean ± standard error of three independent experiments. ^#^ *p* < 0.05 vs. column 2 or column 3; * *p* < 0.05 vs. column 7.

To investigate the role of MCPIP1 in cells, T-REx-293/HA-MCPIP1 and T-REx-293/HA-MCPIP1-D141N cell lines were established by transducing the HA-tagged WT MCPIP1 gene and HA-tagged MCPIP1-D141N mutant gene, respectively, into Tet-on T-REx-293 cells. The D141N mutation in the NYN/DUB domain abolishes its RNase and DUB activities [13]. HA-tagged MCPIP1 was expressed under the presence of doxycycline (Dox), a more-stable tetracycline analog. To determine whether MCPIP1 increased TNF-α-induced apoptosis, we first treated T-REx-293/HA-MCPIP1 cells with 1 μg/mL Dox and then treated them with TNF-α. As shown in Figure 1A,C, TNF-α treatment resulted in 7.2% sub-G_1_ population (apoptotic peak) by flow cytometry and 18.2% cell death by MTT assay; however, overexpression of WT MCPIP1 significantly enhanced the TNF-α-induced sub-G_1_ population to 19.2% and cell death to 41.5%. To further examine whether RNase and DUB activities of MCPIP1 were involved in TNF-α-induced sub-G_1_ population, we used T-REx-293/MCPIP1-D141N cells as control. As shown in Figure 1B,C, overexpression of MCPIP1-D141N mutant resulted in 8.5% sub-G_1_ population and 21.3% cell death under TNF-α treatment. However, overexpression of WT MCPIP1 or the MCPIP1-D141N mutant had no significant effect on the induction of the sub-G_1_ population under Dox treatment for 16 h.

**Figure 2:**
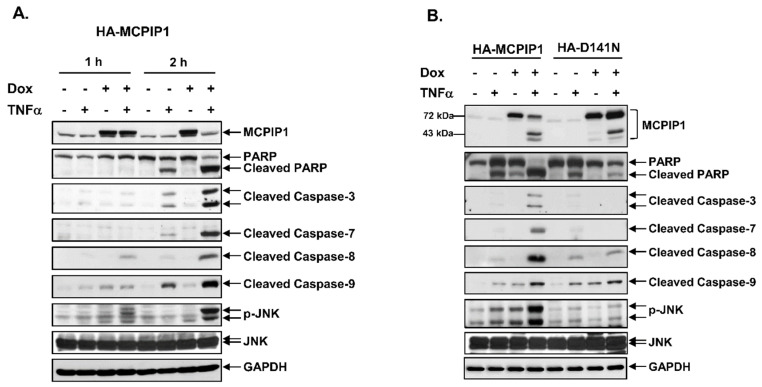
Overexpression of MCPIP1 enhanced TNF-α-activated caspases and JNK. (**A**) T-REx-293 cells with wild-type hemagglutinin (HA)-MCPIP1 were pretreated without or with 1 μg/mL of doxycycline (Dox) for 16 h, and then treated with or without 10 ng/mL TNF-α for 1 and 2 h. (**B**) T-REx-293 cells with wild-type HA-MCPIP1 or the HA-MCPIP1-D141N mutant (HA-D141N) were pretreated without or with 1 μg/mL of Dox for 16 h, and then treated with or without 10 ng/mL TNF-α for 2 h. Total cell lysates were collected, and protein expressions were determined by Western blotting.

Activation of the caspase cascade is an important process during cell apoptosis. To understand whether MCPIP1 could increase activation of the caspase cascade in TNF-α-treated cells, we next examined the activation of caspases-3, -7, -8, and -9 in TNF-α-treated cells by detecting cleaved caspases (the active form) through Western blotting. As shown in Figure 2, TNF-α treatment for 2 h induced the cleavage of poly(ADP-ribose) polymerase (PARP), and caspases-3, -7, -8, and -9 in cells without Dox; however, overexpression of the WT MCPIP1 but not the MCPIP1-D141N mutant enhanced TNF-α-induced cleavage of PARP, and caspases-3, -7, -8, and -9, as well as JNK phosphorylation. Interestingly, both the WT MCPIP1 and MCPIP1-D141N mutant proteins were also cleaved under TNF-α treatment, but overexpression of the MCPIP1-D141N mutant resulted in little cleavage of MCPIP1 (Figure 2B and Figure S1). These results suggested that MCPIP1 enhanced apoptosis and a caspase cascade in TNF-α-treated cells, and cleavage of MCPIP1 might have caused it to lose its activity.

### 3.2. A Pan-Caspase Inhibitor and Caspase-1 Inhibitor Reversed MCPIP1/TNF-α-Mediated Apoptosis and Caspase Activation

**Figure 3:**
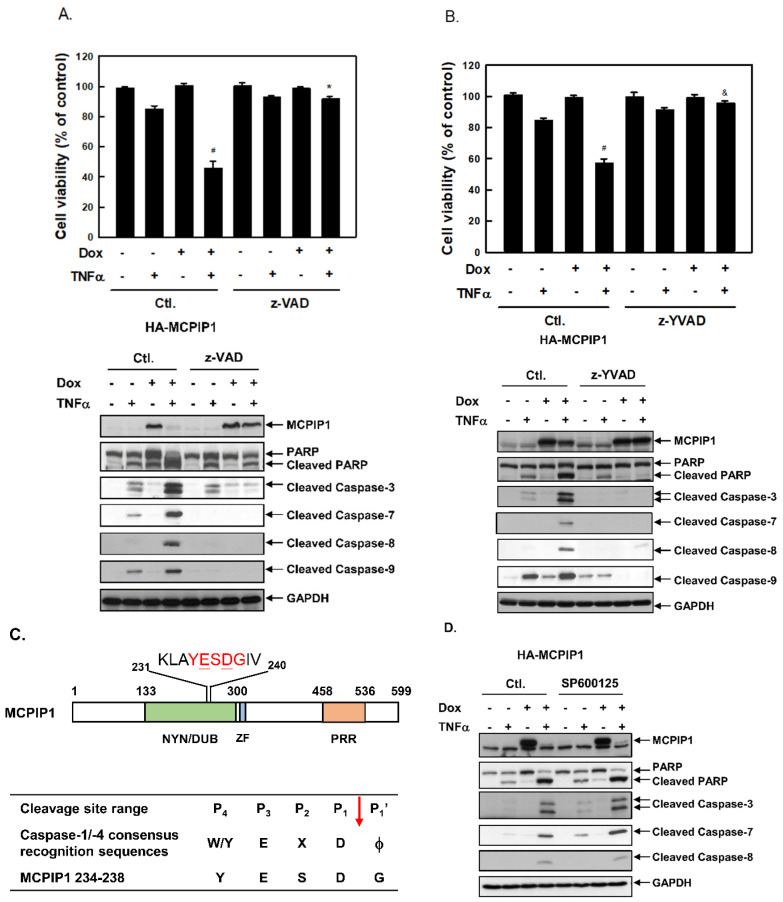
A pan-caspase inhibitor and caspase-1 inhibitor reversed apoptosis and caspase activation in TNF-α-treated cells. T-REx-293 cells with wild-type hemagglutinin (HA)-MCPIP1 were pretreated without or with 1 μg/mL of doxycycline (Dox) for 16 h, and then treated with 10 ng/mL TNF-α for 2 h in the presence or absence of (**A**) 1 μM of the pan-caspase inhibitor, z-VAD-fmk, (**B**) 1 μM of the caspase-1 inhibitor, z-YVAD-fmk, or (**C**) 1 μM of the JNK inhibitor, SP600125. Viable cells were determined by an MTT method. Each value is presented as the mean ± standard error of three independent experiments. ^#^ *p* < 0.05 vs. column 2 or column 3. * *p* < 0.05 vs. column 4 and column 7; ^&^ *p* < 0.05 vs. column 4. Total cellular lysates were collected, and protein expressions were determined by Western blotting. (**D**) Schematic representation of the monocyte chemoattractant protein-1-induced protein 1 (MCPIP1) protein containing Nedd4-BP1, YacP nuclease/deubiquitinase (NYN/DUB), CCCH-type zinc finger (ZF), and proline-rich (PRR) domains as well as putative caspase-1/-4-recognition sequences. The inherent capsase-1/-4 substrate specificity is shown. X is any amino acid, ↓ denotes the site of cleavage, and ϕ refers to G, A, T, S, and N.

To determine the role of caspase activation in MCPIP1/TNF-α-mediated apoptosis, we next examined whether the pan-caspase inhibitor, benzyloxycarbonyl-Val-Ala-Asp(OMe)-fluoromethylketon (z-VAD-fmk), could reverse apoptosis. As shown in Figure 3A, TNF-α treatment resulted in 15.1% cell death, but cell death increased to 54.1% in cells overexpressing MCPIP1. The pan-caspase inhibitor reversed apoptosis and decreased the cleavage of PARP, and caspases-3, -7, -8, and -9 in cells with TNF-α alone or TNF-α plus MCPIP1 overexpression. Interestingly, MCPIP1 cleavage itself also decreased in cells after the addition of the pan-caspase inhibitor. Surprisingly, the caspase-1/-4 inhibitor, benzyloxycarbonyl-Tyr-Val-Ala-Asp(OMe)-fluoromethylketon (z-YVAD-fmk), also reversed apoptosis and decreased the cleavage of PARP and caspases in cells with TNF-α alone or TNF-α plus overexpression of MCPIP1 (Figure 3B).

The caspase-1/-4 inhibitor being able to block cleavage of MCPIP1 indicates that MCPIP1 might be a caspase-1/-4 substrate. Previous studies summarized and reported the consensus substrate specificity of caspases and their inherent substrate preferences [29]. The classic caspase-1/-4 recognition consensus is W/YEXDϕ in the P4 to P1′ positions, where X is any amino acid. The bond between of P1 and P1′ is the caspase cutting site, where uncharged and smaller residues of P1′ (ϕ) are preferred, such as Gly (G), Ala (A), Thr (T), Ser (S), and Asn (N) (Figure 3C). We found that the 234th~238th amino acid sequence of MCPIP1 coincides with the substrate preference of caspases-1/-4, in which ϕ is Gly (Figure 3C). To rule out the possibility that the proteasome may be involved in MCPIP1 cleavage, we used the proteasome inhibitor MG132, and cells treated with TNF-α and Dox were the same as those in Figure 3A,B. MG132 treatment did not block the cleavage of MCPIP1 induced by TNF-α with overexpression of MCPIP1 (Figure S2). These results suggested that MCPIP1 overexpression enhanced TNF-α-induced apoptosis through a caspase cascade, and MCPIP1 cleavage might be mediated through a caspase-1/-4 pathway.

### 3.3. MCPIP1 Downregulated an NF-κB Activation Pathway as Well as Importin α3 and Importin α4 Expressions

TNF-α can induce both a caspase-dependent apoptosis pathway and an NF-κB survival pathway. The NF-κB transcription factor increases expressions of the caspase inhibitors, cIAP and cFLIP, but cFLIP can also be degraded by the TNF-α-JNK-itchy E3 ubiquitin protein ligase (ITCH) signal activation axis. Since MCPIP1 overexpression can increase JNK phosphorylation induced by TNF-α (Figure 2A,B), we next examined whether the JNK inhibitor, SP600125, could decrease caspase activation and apoptosis. Compared to control cells, SP600125 treatment did not change the levels of cleaved caspase-3, -7, or -8 (Figure 3D), and it also did not reverse apoptosis in cells with TNF-α alone or TNF-α plus overexpression of MCPIP1 (data not shown).

**Figure 4:**
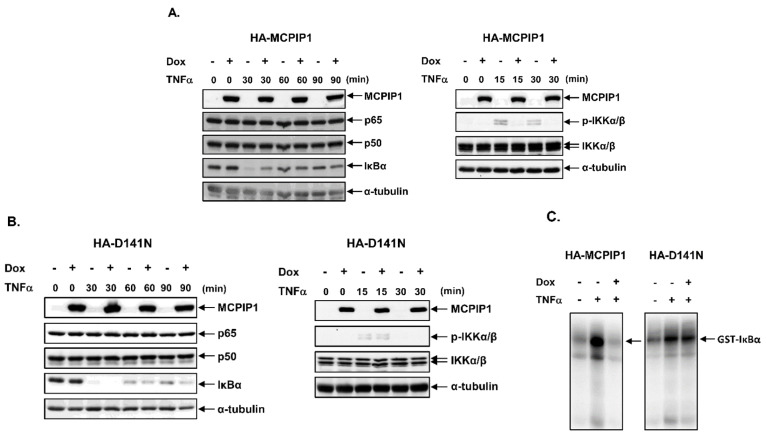

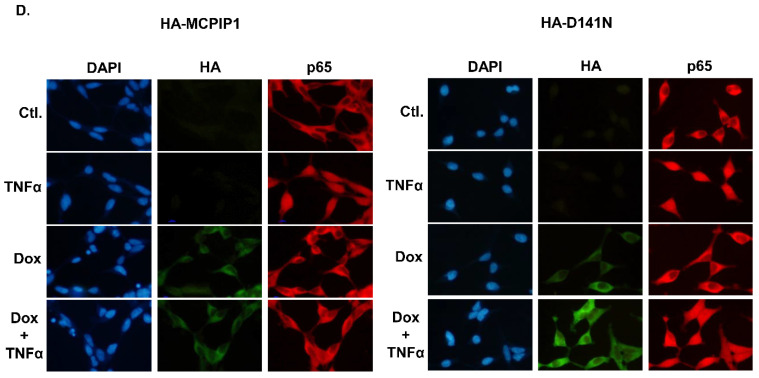
Overexpression of MCPIP1 suppressed NF-κB activation in TNF-α-treated cells. T-REx-293 cells with (**A**) wild-type hemagglutinin (HA)-MCPIP1 or (**B**) the HA-MCPIP1-D141N mutant (HA-D141N) were pretreated without or with 1 μg/mL of doxycycline (Dox) for 16 h, and then treated without or with 10 ng/mL TNF-α for 30~90 min. Total cell lysates were collected and protein expressions were determined by Western blotting. (**C**,**D**) T-REx-293 cells with wild-type HA-MCPIP1 or the HA-MCPIP1-D141N mutant (HA-D141N) were pretreated without or with 1 μg/mL of Dox for 16 h, and then treated with 10 ng/mL TNF-α for (**C**) 15 or (**D**) 30 min. (**C**) Total cell lysates were collected, and IκB kinase (IKK) activity was determined by an in vitro kinase activity assay. (**D**) Cells were fixed and IF staining was performed with NF-κB p65 and HA antibodies, and DAPI was used as a counterstain for DNA.

**Figure 5:**
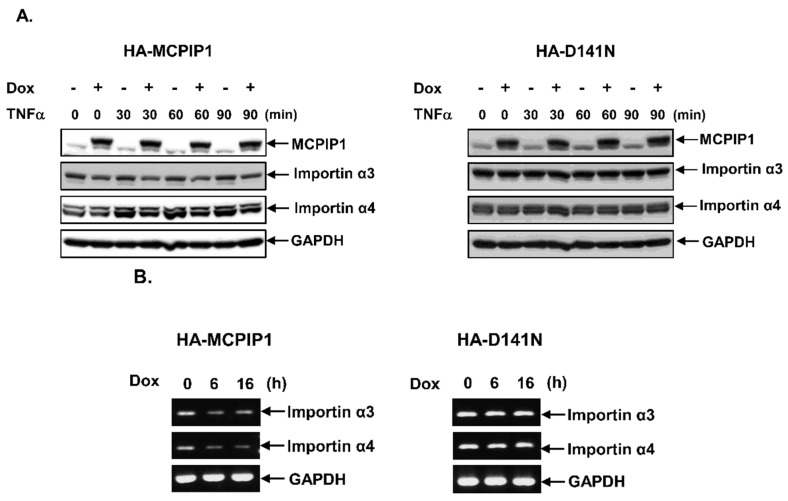
Overexpression of MCPIP1 decreased importin α3 and importin α4 expressions. (**A**,**B**) T-REx-293 cells with wild-type hemagglutinin (HA)-MCPIP1 or the HA-MCPIP1-D141N mutant (HA-D141N) were pretreated without or with 1 μg/mL of doxycycline (Dox) for 16 h, and then treated without or with 10 ng/mL tumor necrosis factor (TNF)-α for 30~90 min. (**A**) Total cell lysates were collected, and protein expressions were determined by Western blotting. (**B**) Total RNA was extracted, and mRNA expression was determined by an RT-PCR.

To confirm that the NF-κB signal pathway was downregulated by MCPIP1, we used Western blotting to detect IκB expression and IKK phosphorylation, an in vitro kinase activity assay to measure IKK activity, IF staining to observe NF-κB translocation, and an EMSA to evaluate NF-κB-binding activity. Overexpression of the WT MCPIP1 but not the MCPIP1-D141N mutant significantly inhibited IκB degradation, IKK phosphorylation (Figure 4A,B), IKK activity (Figure 4C), NF-κB translocation to nuclei (Figure 4D), and NF-κB-binding activity (Figure S3) in TNF-α-treated cells. The transport of NF-κB into nuclei was mainly assisted by importin α3 (also known as karyopherin alpha 4 (KPNA4)) and importin α4 (also known as karyopherin alpha 3 (KPNA3)) [30], so we next investigated whether MCPIP1 could regulate expressions of importin α3 and importin α4. As shown in Figure 5, overexpression of the WT MCPIP1 but not the MCPIP1-D141N mutant downregulated protein and mRNA expressions of both importin α3 and importin α4. These results suggested that MCPIP1’s downregulation of NF-κB activity might be mediated through inhibiting both IKK activity and importin α3 and importin α4 expressions.

### 3.4. MCPIP1 Downregulates Expression of cFLIP, a Caspase-8 Inhibitor

**Figure 6:**
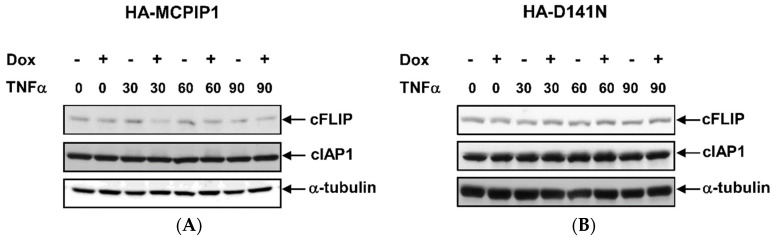
Overexpression of MCPIP1 inhibited cFLIP expression in TNF-α-treated cells. T-REx-293 cells with (**A**) wild-type hemagglutinin (HA)-MCPIP1 or (**B**) the HA-MCPIP1-D141N mutant (HA-D141N) were pretreated without or with 1 μg/mL of doxycycline (Dox) for 16 h, and then treated without or with 10 ng/mL TNF-α for 30~90 min. Total cell lysates were collected, and protein expressions were determined by Western blotting.

Because both cFLIP and cIAP are, respectively, induced to inhibit activation of caspase-8 and caspases-3, -7, and -9 in the TNF-α/NF-κB signal pathway, we next examined whether MCPIP1 could downregulate cFLIP and cIAP1 expressions. As shown in Figure 6, overexpression of the WT MCPIP1 but not the MCPIP1-D141N mutant inhibited cFLIP expression in TNF-α-treated cells. However, neither WT MCPIP1 nor the MCPIP1-D141N mutant affected cIAP expression. These results suggested that MCPIP1-enhanced apoptosis might be mediated through the blocking of NF-κB activation and then the downregulation of cFLIP expression in TNF-α-treated cells.

## 4. Discussion

**Figure 7:**
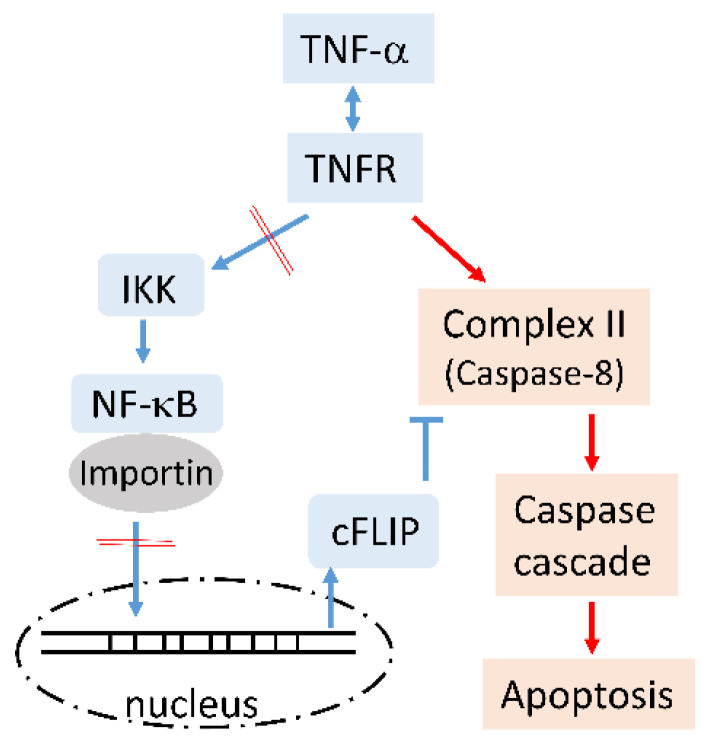
The possible mechanisms of MCPIP1 enhanced TNF-α-induced apoptosis. TNF-α binds to TNF receptor (TNFR) and transduces at least cell survival (blue line) and apoptosis (red line) signals. MCPIP1 may inhibit IKK activation and NF-κB translocation through its deubiquitinase and RNase, respectively, and finally downregulates cFLIP expression. The uninhibited caspase-8 causes cell apoptosis through the caspase cascade pathway.

We used T-REx-293 cells as the experimental model, which were derived from human embryonic kidney 293 cells, and tetracycline was used to control MCPIP1 expression. In addition to expressing WT MCPIP1, another cell line was also used to express the MCPIP1-D141N mutant, which had lost both RNase and deubiquitinase activities. It is known that TNF-α can induce two main pathways: a survival pathway that is mediated through complex-I/TAK1/JNK, IKK/NF-κB/cFLIP, and cIAP, and an apoptotic pathway that is mediated via complex-II, including caspase-8. In this study, MCPIP1 overexpression significantly increased TNF-α-induced apoptosis and caspase activation, and a pan-caspase inhibitor and caspase-1 inhibitor effectively reversed apoptosis and caspase activation. Moreover, the WT MCPIP1 but not the MCPIP1-D141N mutant significantly inhibited IKK activity, and importin α3 and importin α4 expressions, and resulted in the downregulation of NF-κB activation and the limiting of the expression of its downstream caspase-8 inhibitor, cFLIP (Figure 7). The loss of inhibition of IKK activity as well as importin α3 and importin α4 expression were associated with loss of deubiquitinase and RNase activities of MCPIP1-D141N mutant, respectively.

Upon TNF-α binding to its receptor, most cells tend to survive because there are early and late cell death checkpoints [31,32]. The early checkpoint is mainly affected by RIP1 ubiquitination and phosphorylation, which are modified by the TRAF2-cIAP1/2 and LUBAC E3 ligases, and NEMO-IKK-α/β, respectively. Ubiquitinated and phosphorylated RIP1 remains in complex I to transduce survival signals. RIP1 without ubiquitination would form complex IIb with FADD and caspase-8, and then induce apoptosis signals. On the other hand, the late checkpoint is controlled by the transcriptional activity of NF-κB, which inhibit apoptosis by inducing the expression of pro-survival genes, such as cFLIP. When NF-κB is inhibited, the amount of cFLIP is not sufficient to inhibit the caspase-8 activity of complex IIa, which contains at least two other members, FADD and TRADD. Our results found that MCPIP1 suppressed NF-κB activation (Figure 4, Figure 5, Figure 6), suggesting that MCPIP1 might disrupt late checkpoint, and resulted in the blocking of the survival signals. A previous study also demonstrated that MCPIP1 indirectly decreased the RIP ubiquitination by inhibiting TRAF2 signaling [22]. It is possible that MCPIP1 inhibited RIP1 ubiquitination, thus disrupting the early checkpoint and turning on the apoptotic signal in the experimental model of this study. Therefore, the enhancement of apoptosis by MCPIP1 in TNF-α-treated cells might be caused by the simultaneous shutdown of early and late cell death checkpoints.

Interestingly, we found for the first time that MCPIP1 was cleaved during apoptosis, and might be cut by caspases-1/-4. Previous studies showed that many cancer cells exhibit aberrant or constant NF-κB activation, leading to cell survival and resistance to chemotherapy and radiation therapy [33]. Those results suggested that MCPIP1 induction during inflammation not only negatively regulates inflammation but also causes inflammatory cell apoptosis through inhibition of the NF-κB signaling pathway, and finally contributes to the controlling of excessive inflammation. Regardless of the toxic effects of cancer patients caused by TNF-α treatment, MCPIP1 upregulation might help to kill tumor cells and reduce the amount of TNF-α treatment needed.

It is well known that importin α family proteins are involved in the nuclear translocation of many transcription factors. Studies also found that importin α3 and importin α4 increased in a variety of tumor tissues, and that the downregulation of importin α3 and importin α4 inhibited tumor cell proliferation, migration, and invasion. For example, importin α4 is increased in colorectal cancer, and importin α3 is elevated in head and neck squamous cell carcinoma and papillary thyroid cancer [34,35,36]. Therefore, inhibition of mRNA expressions of both importin α3 and importin α4 by MCPIP1 (Figure 5) might limit the translocation of NF-κB and other molecules to nuclei. Moreover, the antitumor effects of MCPIP1 might be mediated through the suppression of importin α3 and importin α4 expressions.

MCPIP1 is a transcription factor and an RNA-binding protein (RBP) with endogenous RNase activity. Therefore, MCPIP1 can regulate mRNA expression at both the transcriptional and post-transcriptional levels. Previous studies also demonstrated that MCPIP1 specifically recognizes SLs present in the 3′UTR of certain proinflammatory mRNAs, including TNF-α, IL-2, and IL-17 [37], in an AU-rich element (ARE)-independent manner. Other RBPs, such as tristetraprolin (TTP) [38], ARE RNA-binding protein 1 (AUF1), and human antigen R (HuR, also known as ELAV1) were found to regulate the mRNA stability of certain proinflammatory genes through binding of the ARE structure [39,40]. The mRNA 3′UTRs of both importin α3 and importin α4 were identified—using the online software RNAfold (http://rna.tbi.univie.ac.at//cgi-bin/RNAWebSuite/RNAfold.cgi accessed on 31 March 2021) and AREsite (http://rna.tbi.univie.ac.at/AREsite2/welcome accessed on 31 March 2021)—to be potentially capable of forming SLs and ARE sites. In this study, we found that MCPIP1 did not enter the nuclei of cells with or without TNF-α treatment by IF staining (Figure 4D), and the MCPIP1-D141N mutant (without RNase) did not affect importin α3 or importin α4 expression, suggesting that the MCPIP1 downregulation of importin α3 and importin α4 expression is possibly mediated, at the posttranscriptional level, by binding to the SLs of the 3′UTR of importin α3 and importin α4 mRNAs.

Previous studies demonstrated that MCPIP1 negatively regulates NF-κB activation and JNK phosphorylation in LPS- and IL-1β-treated macrophages [22]. On the contrary, we found that MCPIP1 decreased JNK phosphorylation in TNF-α-treated cells (Figure 2B). The controversial role of JNK may further result in degradation of the caspase-8 inhibitor, cFLIP, or an increase in antiapoptotic gene expression via c-Jun/AP-1 activation [41]. Nevertheless, the JNK inhibitor, SP600125, did not change the cleavage of PARP, or caspase-3, -8, or -9, indicating that JNK was not involved in TNF-α/MCPIP1-mediated apoptosis.

In this study, MCPIP1 was found to enhance activation of the TNF-α-induced caspase cascade, including caspases-3, -7, -8, and -9. Unexpectedly, MCPIP1 itself was also cleaved (Figure 2B), and the cleavage could be blocked by the caspase-1/-4 inhibitor, z-YVAD-fmk (Figure 3B). By searching and aligning amino acid sequences of MCPIP1, we located a caspase-1/-4 consensus recognition sequence in the 234th~238th residues of the MCPIP1 NYN/DUB domain. This evidence further confirmed that MCPIP1 is indeed a substrate of capsases-1/-4. Generally, MCPIP1 is upregulated upon stimulation with proinflammatory cytokines, such as IL-1β, TNF-α, and LPS [42], and then negatively feeds back to inhibit excessive inflammation. Cleavage of MCPIP1 by caspases-1/-4 might cause it to lose its deubiquitinase and RNase activities and then restore the inflammatory ability of immune cells. However, it cannot be ruled out that the deubiquitinase and RNase activities of cleaved MCPIP1 were increased compared to full-length MCPIP1. It should be noted that the MCPIP1-D141N mutant did not enhance caspase activities in TNF-α-treated cells, so it was less cleaved than WT MCPIP1. T-REx-293 cells used in this study were originally from human embryonic kidney (HEK) 293 cells. Although we did not detect the presence of caspases-1/-4 by Western blotting in T-REx-293 cells and other studies also indicated that 293 cells expressed very little caspases-1/-4 [43,44], the caspase-1/-4 inhibitor, z-YVAD-fmk, significantly reduced apoptosis caused by TNF-α/MCPIP1 in this study, and caspase-1 also induced apoptosis via the Bid/caspase-9/caspase-3 axis from earlier studies [45].

Previous studies have demonstrated that the CARMA1-BCL10-MALT1 (CBM) complex plays an important bridging role between the T or B cell antigen receptor (TCR/BCR) proximal signaling and NF-κB and JNK activation. After TCR activation, MCPIP1 could be cleaved at Arg111 by MALT1 paracaspase activity, and the MALT1-mediated cleavage of MCPIP1 was associated with the restoration of T-cell effector gene expression [46] and Th17 differentiation [47]. However, TNF-α-activated NF-κB may be dependent on [48], or independent of [49], MALT1 in murine embryonic fibroblasts. It could not be excluded that MALT1 was involved in the cleavage of MCPIP1 in TNF-α-treated T-REx-293 cells, but further experiments are needed to obtain a clear answer.

## 5. Conclusions

In conclusion, we found that MCPIP1 enhanced TNF-α-induced caspase activities and apoptosis, and those could be reversed by a caspase-1/-4 inhibitor. The increase in caspase activities and apoptosis by MCPIP1 might be mediated through downregulation of the NF-κB pathway, including the inhibition of IKK activity and importin α3 and importin α4 expression, and finally the suppression of cFLIP expression. In addition, we identified for the first time that MCPIP1 is a caspase-1/-4 substrate, and that the cleavage of MCPIP1 may play an important role in caspase-1/-4-initiated apoptosis.

## Data Availability

The data presented in this study are available within the article.

## Supplementary Materials

The following are available online at https://www.mdpi.com/article/10.3390/biology10070655/s1, Figure S1: Overexpression of the MCPIP1-D141N mutant did not cause cleavage of MCPIP1 in TNF-α-treated cells, Figure S2: A proteasome inhibitor did not suppress MCPIP1 cleavage in TNF-α-treated cells, Figure S3: Overexpression of MCPIP1 inhibited NF-κB binding activity in TNF-α-treated cells.
