# Supplementary material for: MCPIP1 Enhances TNF-α-Mediated Apoptosis through Downregulation of the NF-κB/cFLIP Axis
Source: Biology (Basel). 2021 Jul 12;10(7):655. doi: 10.3390/biology10070655 (PMC8301320; doi:10.3390/biology10070655)
Supplement: Supplementary file 1 [file biology-10-00655-s001.zip › biology-1261273-supplementary.pdf]

biology MDPI

Supplementary Materials

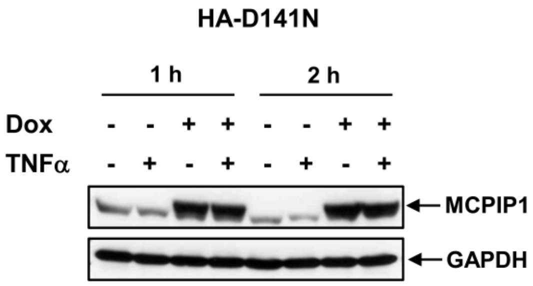

Figure S1. Overexpression of the MCPIP1-D141N mutant did not cause cleavage of MCPIP1 in TNF-α-treated cells. T-REx-293 cells with the HA-MCPIP1-D141N mutant (HA-D141N) were pretreated without or with 1 μg/ml of doxycycline (Dox) for 16 h, and then treated with or without 10 ng/ml TNF-α for 1 and 2 h. Total cell lysates were collected, and protein expressions were determined by Western blotting.

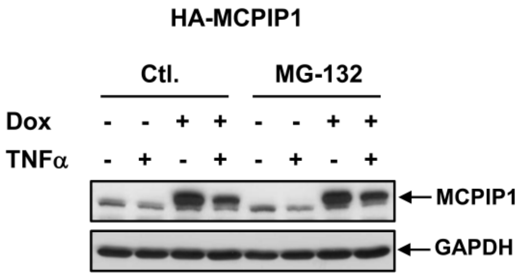

Figure S2. A proteasome inhibitor did not suppress MCPIP1 cleavage in TNF-α-treated cells. T-REx-293 cells with wild-type hemagglutinin (HA)-MCPIP1 (HA-MCPIP1) were pretreated without or with 1 μg/ml of doxycycline (Dox) for 16 h, and then treated with or without 10 ng/ml TNF-α for 2 h. Total cell lysates were collected, and protein expressions were determined by Western blotting.

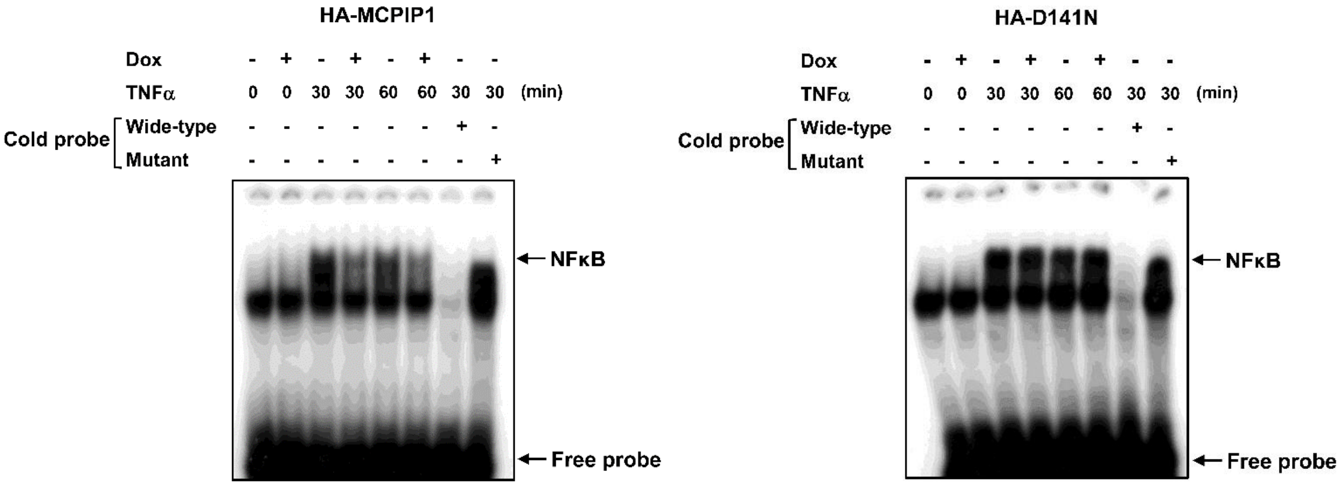

Figure S3. Overexpression of MCPIP1 inhibited NF-κB binding activity in TNF-α-treated cells. T-REx-293 cells with wild-type hemagglutinin (HA)-MCPIP1 or the HA-MCPIP1-D141N mutant (HA-D141N) were pretreated without or with 1 μg/ml of doxycycline (Dox) for 16 h, and then treated without or with 10 ng/ml TNF-α for 30~60 min. Nuclear extracts were prepared, and an electrophoretic mobility shift assay (EMSA) was carried out as described in "Methods and Materials". The specificity of NF-κB complex formation is shown.
